# Developmental brain abnormalities and acute encephalopathy in a patient with myopathy with extrapyramidal signs secondary to pathogenic variants in MICU1

**DOI:** 10.1002/jmd2.12114

**Published:** 2020-03-20

**Authors:** Katelynn M. Wilton, Joel A. Morales‐Rosado, Duygu Selcen, Karthik Muthusamy, Sarah Ewing, Katherine Agre, Katherine Nickels, Eric W. Klee, Mai‐Lan Ho, Eva Morava

**Affiliations:** ^1^ Medical Scientist Training Program, Mayo Clinic Alix College of Medicine Mayo Clinic Rochester Minnesota USA; ^2^ Center for Individualized Medicine Mayo Clinic Rochester Minnesota USA; ^3^ Department of Health Science Research, Division of Biomedical Statistics and Informatics Mayo Clinic Rochester Minnesota USA; ^4^ Department of Neurology Mayo Clinic Rochester Minnesota USA; ^5^ Department of Clinical Genomics Mayo Clinic Rochester Minnesota USA; ^6^ Department of Radiology Nationwide Children's Hospital Columbus Ohio USA

**Keywords:** acute disseminated encephalomyelitis, genetic, MICU1, MICU1 deficiency, mitochondria, MPXPS

## Abstract

Mitochondria play a variety of roles in the cell, far beyond their widely recognized role in ATP generation. One such role is the regulation and sequestration of calcium, which is done with the help of the mitochondrial calcium uniporter (MCU) and its regulators, MICU1 and MICU2. Genetic variations in MICU1 and MICU2 have been reported to cause myopathy, developmental disability and neurological symptoms typical of mitochondrial disorders. The symptoms of MICU1/2 deficiency have generally been attributed to calcium regulation in the metabolic and biochemical roles of mitochondria. Here, we report a female child with heterozygous MICU1 variants and multiple congenital brain malformations on MRI. Specifically, she shows anterior perisylvian polymicrogyria, dysmorphic basal ganglia, and cerebellar dysplasia in addition to white matter abnormalities. These novel findings suggest that MICU1 is necessary for proper neurodevelopment through a variety of potential mechanisms, including calcium‐mediated regulation of the neuronal cytoskeleton, Miro1‐MCU complex‐mediated mitochondrial movement, or enhancing ATP production. This case provides new insight into the molecular pathogenesis of MCU dysfunction and may represent a novel diagnostic feature of calcium‐based mitochondrial disease.

**SYNOPSIS**

We describe a patient with myopathy with extrapyramidal signs secondary to compound heterozygous variant in Mitochondrial Calcium Uptake 1 (MICU1) presenting with a novel phenotype of diffuse brain malformations, indicative of disrupted neuronal development, and associated with seizures and encephalopathy.

## 1 INTRODUCTION

Mitochondria are required for critical cellular functions, including apoptosis, metabolism, and calcium dynamics. In most mitochondrial diseases, symptoms arise from dysregulation of the two cell types with the highest metabolic demand: muscle cells (proximal myopathy, dysphagia, respiratory insufficiency, and cardiac disease) and neurons (developmental delay, ophthalmoplegia, epilepsy, stroke‐like episodes).^1^ Most patients present with an unpredictable subset of these symptoms, as mitochondrial disease produces diverse phenotypes, even among family members.^2^ The exact etiology of this heterogeneity is unclear, although mitochondrial heteroplasmy, organ‐specific mitochondrial distributions, and interaction between nuclear and mitochondrial encoded proteins likely contribute.^3^

Biallelic pathogenic variants in one nuclear‐encoded protein, mitochondrial calcium uptake 1 (MICU1) have recently been described to cause myopathy with extrapyramidal signs (MPXPS). MPXPS has been reported in 41 cases (Table S1) and presents with myopathy, learning disability, and extrapyramidal movement disorder.^4^, ^5^, ^6^, ^7^, ^8^, ^9^, ^10^, ^11^, ^12^ The MICU1 protein regulates calcium influx into mitochondria through interaction with the mitochondrial calcium uniporter (MCU). At baseline, MCU continuously moves calcium into the mitochondria. The MICU1 protein is a calcium‐sensor for MCU, allowing decreased calcium uptake when cytoplasmic calcium is low. Although mitochondrial calcium homeostasis plays diverse roles in cellular signaling,^13^ mitochondrial metabolism,^13^ programmed cell death^13^, ^14^, ^15^ and cell migration,^16^, ^17^, ^18^, ^19^ previously‐described phenotypes of MPXPS have been largely attributed to biochemical dysregulation and impaired ATP production.^20^, ^21^

Here we report a female child with compound heterozygous variants in MICU1, who presents with typical symptoms of mitochondrial disease, including myopathy, ataxia, developmental delay, and generalized seizures,^22^ without an elevated lactate level. In addition to white matter changes, her magnetic resonance imaging scans showed multifocal brain malformations including anterior perisylvian polymicrogyria, dysmorphic basal ganglia, and cerebellar dysplasia. These findings have not been previously reported in MPXPS.

## 2 CASE PRESENTATION

**Figure 1:**
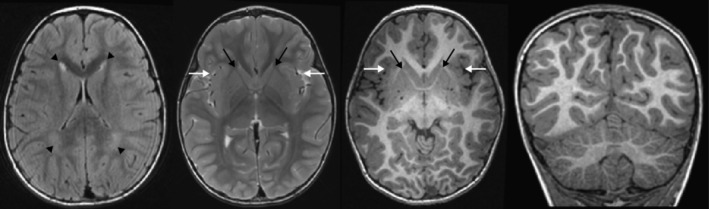
Baseline brain malformations in a patient with MPXPS. Baseline MRI at age 3 years shows patchy periventricular white matter signal changes (black arrowheads), anterior perisylvian polymicrogyria (white arrows), dysmorphic basal ganglia with hypoplastic anterior limbs of the internal capsules (black arrows), and mild cerebellar dysplasia with broad palisaded folia

The patient was born at term from an uncomplicated pregnancy into a family with no known family history of childhood developmental delay, neurologic conditions, genetic disease, or consanguinity. At birth, she was 3.5 kg with a length of 48.3 cm and a head circumference of 35.6 cm. Her newborn screening tests, including hearing tests, were within normal limits. She experienced an RSV infection at 6 months, sat at 9 months, and was able to scoot and roll over at 1 year of age. At 3 years, she began speaking in sentences but also showed delays in fine motor control. Limited magnetic resonance imaging of her brain (Figure 1) was performed using a general protocol with basic sequences. The study was initially reported as normal, but in retrospect showed subtle multifocal brain malformations and white matter abnormalities. Specifically, there was bilateral anterior perisylvian polymicrogyria, dysmorphic basal ganglia with hypoplastic anterior limbs of the internal capsules, mild cerebellar dysplasia with broad palisaded folia, and patchy periventricular white matter signal changes. She later developed amblyopia at 4 years of age.

**Figure 2:**
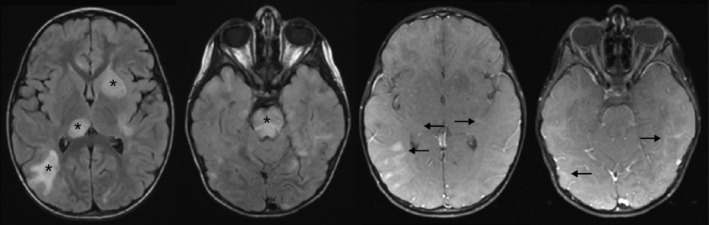
Acute encephalopathy in a patient with MPXPS and baseline structural brain abnormalities. At age 5 years, during an acute encephalopathic episode, MRI shows multifocal confluent edema (FLAIR, asterisks, left panels) and patchy enhancement (T1 post contrast, black arrows, right panels) in the subcortical and deep white matter, optic nerves, basal ganglia, brainstem and cerebellum

At 5 years old, she had pneumonia, which was treated with cefdinir without improvement. She was subsequently switched to azithromycin for potential mycoplasma pneumonia. She began to recover but then developed altered mental status, increased ataxia, and stiffened gait. Her laboratory evaluations were grossly normal except for leukocytosis, increased CSF protein, and elevations in specific amino acids (valine and lysine). Mycoplasma pneumonia serologies, chest radiograph, CSF oligoclonal bands, and paraneoplastic antibody panel were unremarkable. Magnetic resonance imaging of her brain was performed and showed multifocal confluent areas of edema and patchy enhancement in the subcortical and deep white matter, optic nerves, basal ganglia, brainstem, and cerebellum (Figure 2), suggestive of acute disseminated encephalomyelitis (ADEM) or other parainfectious syndromes. She was treated with a 5‐day course of IV methylprednisolone, which resulted in significant improvement. One month later, she showed some remnant ataxia and slight clumsiness on fine motor skills but had otherwise returned to her clinical baseline.

**Figure 3:**
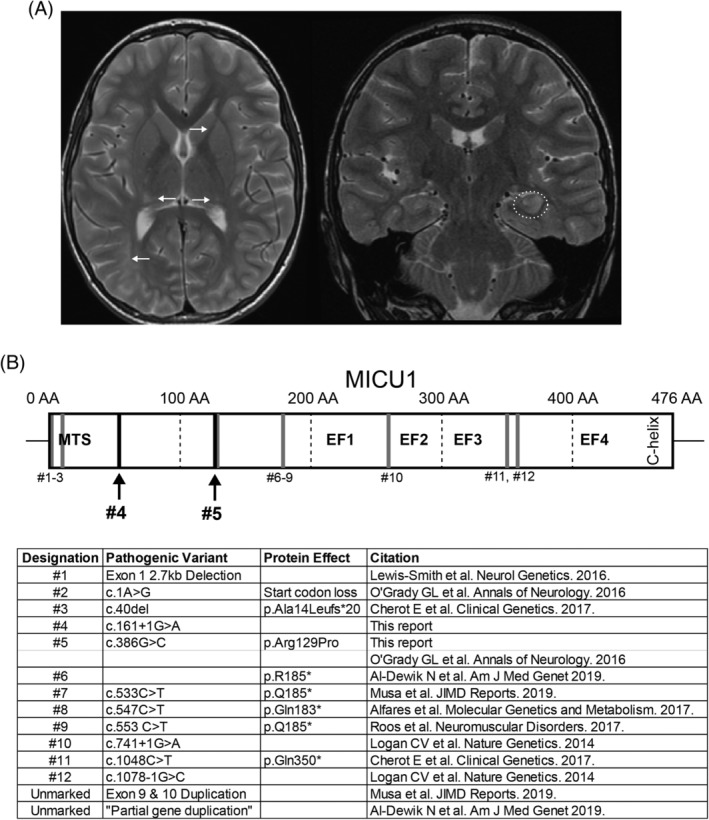
Neurological and genetic presentation of seizures in a patient with MPXPS and baseline structural brain abnormalities: A, At age 12 years, new seizures with epilepsy protocol MRI shows chronic encephalomalacia from the prior parainfectious syndrome (white arrows) and acute postictal changes in the left hippocampus (dotted oval). B, Reported pathogenic variations associated with MPXPS. Previously reported variations are roughly noted, in dark grey lines. Pathogenic variations reported in the described patient are shown in prominent dark lines designated #4 and #5

Seven years later, the patient developed new tonic‐clonic seizures with secondary generalization. Her seizures involved initial eye and limb twitching with decreased responsiveness. She then proceeded to become stiff and proceed into a tonic‐clonic seizure. She experienced status epilepticus and an apneic episode with O_2_ desaturation. Although her seizures were not directly observed on conventional or computer‐assisted prolonged video electroencephalography (EEG), her EEG pattern did show abnormalities, including frequent left‐lateralized periodic discharges over left temporal head region, with occasional multifocal left temporal, midline and right frontal sharp waves, a mild degree of nonspecific diffuse slowing of background activity and excessive fast activity in the background. She continued to have elevated creatinine kinase, but a normal lactate level. Magnetic resonance imaging (Figure 3A) using high‐resolution epilepsy sequences confirmed her congenital brain malformations, now with superimposed chronic encephalomalacia from the prior parainfectious syndrome, and acute postictal changes in the left hippocampus.

On examination, the patient has abnormal narrow facies with a short prominent upturned nose, epicanthal folds, and hypertelorism. At 12 years of age, she was prominently hyperreflexic, and showed clonus on the left side.

## 3 GENETIC INVESTIGATION

The patient's encephalopathy and myopathy were investigated for a genetic cause. Chromosome analysis showed karyotype of 46XX with no gross chromosomal abnormalities. An epilepsy deletion/duplication panel was completed at GeneDx using an exon‐level oligo array CGH (ExonArrayDx). Data analysis was performed with respect to genes of interest and analyzed in comparison to the human genome build GRCh37/USChg19. This analysis, including 96 genes, showed no pathogenic variants or variants of uncertain significance (VUS). Further analysis was obtained via GeneDx EpiXpanded panel, using a proprietary capture method for Next‐Generation Sequencing with CNV calling. The enriched genes were sequenced bidirectionally using an Illumina platform and aligned to the human genome build GRCh37/UCSChg19. XomeAnalyzer was used to analyse the data. Over 1400 genes were analyzed, with no pathogenic variants revealed. Whole‐genome sequencing was completed at GeneDx using similar methods to the epilepsy deletion/duplication panel described above. Whole exome sequencing revealed two variants in the MICU1 nuclear‐encoded gene, each defined about transcript variant 1 (NM_006077.3). Of note, this gene was not included in the GeneDx EpiXpanded Panel. The first (c.161 + 1G > A), was a pathogenic maternally inherited variant. This splicing variant is rare as reported in low frequency (0.0018%; 5/275750 alleles) primarily in the non‐Finnish European population (gnomAD v2.1.1^23^) with no homozygotes observed. This variant destroys the second exon canonical donor site most likely causing a loss of function effect via intron retention or exon skipping. Alternatively, a strong exonic splice site is predicted by SpliceAI to occur 23 nucleotides upstream and its use would also lead to an out‐of‐frame transcript. The second (c.386G > C), a likely pathogenic variant, was paternally inherited. This variation is predicted to result in a p.R129P missense mutation at the protein level. This variant is also present in population databases at a low frequency (0.0071%; 20/279944 alleles) with no homozygotes reported. Residue 129 is highly conserved across orthologues and in silico predictions suggest this variant to be deleterious.

## 4 DISCUSSION

MPXPS, secondary to pathogenic variants in MICU1, can present with myopathy, developmental delay, and extrapyramidal symptoms. This case highlights two other clinical manifestations: encephalopathy (novel) and seizures (reported previously in Reference 10). Interestingly, in this case, MRI demonstrated multiple brain malformations indicative of diffusely disrupted neuronal development. The patient's MICU1 variants are predicted to be pathogenic by in silico analysis (including analysis for protein prediction, uniqueness and evolutionary conservation via SIFT/Polyphen 2/M‐CAP/CADD) and are not in close proximity to previously reported pathogenic variants (Figure 3B).

The MICU1 protein contains a mitochondrial N‐terminal targeting sequence, four EF‐hand calcium‐binding domains (two of which are functional) and a carboxy‐terminal C‐helix.^24^ The reported pathogenic variants in MICU1 occur in diverse regions (Figure 3B). Of note, both variants, in this case, are present at low frequencies with no reports of homozygotes in the general population. Most pathogenic variants in MICU1 have been reported as loss‐of‐function variants^6^, ^8^, ^9^, ^12^ except for the second variant reported in this case, (c.386G > C). The nonconservative arginine to proline substitution, occurring in a stretch of highly conserved basic side chain residues, likely disrupts the secondary structure or negatively affects the folding kinetics. Furthermore, this variant occurs near residues 99 to 102 that are critical for the EMRE/SMTDT1 interaction and downstream function.^25^

Seizures and encephalopathy are common in mitochondrial myopathies.^26^, ^27^, ^28^, ^29^ The underlying pathomechanism for encephalopathic episodes in patients with mitochondrial myopathies is not clear.^30^ MR findings during the patient's encephalopathic presentation (Figure 2) were reminiscent of acute disseminated encephalomyelitis (ADEM, Figure 3A). However, it has been proposed, in a POLG‐linked mitochondrial myopathy, that the neuroinflammation might actually be secondary to the mitochondrial defect, or that these two possible causes could interact or overlap.^30^ Although these ADEM or ADEM‐like encephalopathies are relatively rare in mitochondrial disease, they represent a significant medical burden. An improved understanding of the pathogenesis may lead to better treatments and potential prevention of damage to the brain. In this case, for instance, the patient did not experience seizures until she was 12 years old, 7 years after her encephalopathy. Based on neurologic examination and EEG, the findings from her prior encephalopathy did not fully explain her subsequent epileptic presentation.

This case represents the first description of brain structural abnormalities in a patient with MPXPS. Specifically, this patient showed multiple brain malformations compatible within utero disruption of neuronal development. These findings may represent a novel manifestation or potentially under‐recognized sign of MPXPS and may explain many of the MPXPS‐associated neurological signs and symptoms. Past studies have focused on the biochemical impact of MICU1 pathogenic variants, but little attention has been paid to the potential effects on neuronal development and migration. For instance, cytoplasmic calcium flux is greatly impacted by mitochondrial calcium stores and release^31^ and is responsible for cell movement via changes in the cytoskeleton.^13^, ^32^, ^33^, ^34^, ^35^ A second hypothesis would be that MICU1 is needed for the movement of mitochondria, a prerequisite for neuronal growth and extension.^36^, ^37^ The adaptor protein that links mitochondria to the motor proteins is Miro1, which requires the MCU complex to bind mitochondria.^38^ If the absence of MICU1 prevents this interaction, mitochondria may not be properly localized, leading to abnormal localization of dendrites, axons, and potentially cell bodies. A third possibility is that neuronal development could simply be altered secondary to the decreased production of ATP with decreased global energy stores. Interestingly, the brain malformations seen in this patient are analogous to those seen with defects in cytoskeletal proteins, including tubulinopathies^39^, ^40^, ^41^, ^42^; extracellular matrix proteins, including congenital muscular dystrophies^11^; and inborn errors of metabolism including peroxisomal disorders,^43^, ^44^, ^45^ PDHc deficiency^46^, ^47^ and glutaric acid deficiency,^48^, ^49^ further supporting these potential hypotheses. Normal neuronal generation, migration, and differentiation rely on the maintenance of cytoskeletal architecture as well as appropriate energy stores. Thus, both mechanical disruptions of scaffolding and energetic disruptions of metabolism could converge on the final common pathway of disrupted neuronal development, occurring over an extended time period during gestation.

In addition, the presence of brain malformations may be able to help classify the diverse phenotypes seen in MPXPS. For instance, some patients experience only myopathy, whereas others experience encephalopathy, seizures, and severe learning disabilities. It is possible that the degree of structural abnormalities seen in the brain may correlate with or predict future neurologic symptoms. In our patient, clinical seizures were correlated with her polymicrogyria on MRI, encephalopathy with white matter changes, clonus with basal ganglia dysmorphism, and ataxia with cerebellar dysplasia.

## 5 CONCLUSION

Biallelic pathogenic variants in MICU1 cause MPXPS, which classically presents with myopathy, developmental delay, and extrapyramidal signs. Clinical features have previously been attributed to changes in mitochondrial metabolism secondary to altered calcium homeostasis. In this patient's course, additional episodic symptoms were present, including encephalopathy and seizures. The finding of multiple brain malformations on MRI suggests that MICU1 may be necessary for neuronal development and migration. Although the exact mechanism remains unclear, future studies will hopefully clarify whether structural abnormalities are a diagnostic feature of MPXPS and their predictive value for neurological outcomes.

## CONFLICT OF INTEREST

W.K.M., M.‐R.J.A., S.D., M.K., E.S.A., A.K., N.K., K.E.W., H.M.L., and M.‐K.E. declare they have no conflict of interest.

## AUTHOR CONTRIBUTIONS

W.K.M., M.‐R.J.A.,W. K.M., M.‐R.J.A., and E.S.A. drafted the article. W.K.M., M.‐R.J.A., E.S.A., and M.‐K.E. collected the data, performed variant/data analysis, and interpretation. M.K. and H.M.L. reviewed and interpreted the radiologic findings. N.K. reviewed and interpreted the E.E.G. findings. W.K.M., M.‐R.J.A., W.K.M., M.‐R.J.A., S.D., M.K., E.S.A., A.K., N.K., K.E.W., H.M.L., and M.‐K.E., performed critical review of the final manuscript and approved the final version of the manuscript.

## INFORMED CONSENT

Informed consent was obtained from the patient and family members for inclusion of clinical descriptions and images.

## Supporting information


**Supplemental Table 1** **Reported Cases, Genotypes and Phenotypes of MPXPS Secondary to Pathogenic Variants in MICU1**. OA ‐ Optic atrophy, CTS ‐ cataracts, NYS ‐ nystagmus, PTS ‐ ptosis, HMP ‐ hypermetropia, ASM ‐ Astigmatism, n/a ‐ not available, or not reported
