# Supplementary material for: Developmental brain abnormalities and acute encephalopathy in a patient with myopathy with extrapyramidal signs secondary to pathogenic variants in MICU1
Source: JIMD Rep. 2020 Mar 20;53(1):22–8. doi: 10.1002/jmd2.12114 (PMC7203647; doi:10.1002/jmd2.12114)
Supplement: Supplementary file 1 — Supplemental Table 1 Reported Cases, Genotypes and Phenotypes of MPXPS Secondary to Pathogenic Variants in MICU1. OA ‐ Optic atrophy, CTS ‐ cataracts, NYS ‐ nystagmus, PTS ‐ ptosis, HMP ‐ hypermetropia, ASM ‐ Astigmatism, n/a ‐ not available, or not reported [file JMD2-53-22-s001.docx]

|  | **This report** | **O’Grady et al., 2016 (n = 1)** | **Cherot et al., 2018 (n = 1)** | **Musa et al., 2017**  **(n=13)** | | **Alfares et al., 2017**  **(n = 1)** | **Al-Dewik et al. 2019 (n=6)** | | **Roos et al., 2017 (n=1)** | **Logan et al., 2014 (n = 15)** | | **Lewis-Smith et al., 2016**  **(n = 2)** | |
| --- | --- | --- | --- | --- | --- | --- | --- | --- | --- | --- | --- | --- | --- |
| **PMID:** |  | 27159402 | 28708303 | 29721912 | | 28454995 | 30919572 | | Abstract | 24336167 | | 27123478 | |
| **Identifier** | Our patient | Patient 103 | Patient 22 | Family 7 | Family 1-6  Family 8-9 | Case 84 | Patient 7 | Patients 21, 24, 68, 104, 129, 214 | Patient 1 | Pedigree ID 1-8b | Pedigree LUMC, UMCU | IV:3 | IV:6 |
| **Age and gender**  **(age of onset)** | 12y F | 8y M | 4y M | 4y F | 4 F  (2-11yo)  8 M  (4-23yo) | Not specified | Not specified | Not specified | 3y F | 7 F  (18mo – 3y)  4 M  (18mo – 5y) | 2 M  (11mo, 28mo)  2 F (6-8y) | 9y female | 12y M |
| **cDNA (NM_001195518.1) & Protein Change** | c.386G>C  p.Arg129Pro | c.386G>C  p.Arg129Pro | c.40del  p.Ala14Leufs*20 | c.533C>T  p.Q185* | c.533C>T  p.Q185* | c.547C>T  p.Gln183* | c.553 C>T  p.Q185* | c.553 C>T  p.Q185* | p.R185* | c.1078-1G>C | c.741+1G>A | Exon 1  2.7kb deletion | |
|  | c.161+1G>A  p.? | c.1A>G  Start loss | c.1048C>T  p.Gln350* | Exon 9 and 10 dup (het) |  |  | Partial Gene Duplication |  |  |  |  |  |  |
| **Microcephaly** | - | n/a | n/a | n/a | n/a | n/a | n/a | n/a | n/a | + (5/11) | - | n/a | n/a |
| **Short stature** | - | n/a | n/a | + | + (5/9) | n/a | n/a | + (1/6) | n/a | + (3/11) | + (1/4) | + | - |
| **Poor growth** | - | n/a | n/a | - | + (4/9) | n/a | n/a | n/a | n/a | n/a | n/a | + | - |
| **Muscle**  **weakness** | + | Proximal weakness | + | + | + (7/12) | n/a | n/a | + (2/6) | + | Proximal weakness (4/11) | + (2/4) | - | + |
| **Opthalmologic findings** | Hyperopia, amblyopia | n/a | n/a | n/a | n/a | n/a | n/a | n/a | n/a | + (3/11)  OA, NYS, PTS | + (3/4) | - | OA, CTS, NYS |
| **Extrapyramidal signs** | + | + | + | - | + (5/12) | + | n/a | + (1/6) | + | + (8/11) | + (3/4) | - | - |
| **Neuropathy** | - | n/a | + | n/a | n/a | n/a | n/a | n/a | n/a | + (1/7) | n/a | - | n/a |
| **Abnormal gait** | + | n/a | n/a | + | + (3/10) | n/a | n/a | n/a | + | + (1/11) | + (2/4) | Upon activity or infections | |
| **Seizures** | + | n/a | n/a | n/a | + (4/12) | n/a | n/a | n/a | n/a | n/a | n/a | - | - |
| **Intellectual and/or learning difficulties** | + | + Moderate | + Mild | - | + (10/11) | n/a | n/a | + (3/6) | + | + (10/11) | + (4/4) | - | + |
| **Developmental delay** | + | + | n/a | + | + (12/12) | n/a | + | + (2/6) | + | + (11/11) | + (2/4) | - | + |
| **Speech delay** | + | n/a | n/a | n/a | + (10/10) | n/a | n/a | n/a | n/a | + (6/11) | + (1/4) | n/a | n/a |
| **Facial dysmorphisms** | + | n/a | n/a | - | + (3/12) | n/a | n/a | + (2/6) | n/a | n/a | n/a | n/a | + |
| **Hepatomegaly** | - | n/a | n/a | n/a | + (2/7) | n/a | n/a | n/a | n/a | n/a | n/a | n/a | n/a |
| **Ambulation difficulties** | + | n/a | n/a | + | + (6/10) | n/a | n/a | n/a | + | +(3/11) | + (4/4) | Upon activity or infections | |
| **MM biopsy changes** | n/a | Type 1 fiber predominance | n/a | n/a | n/a | n/a | n/a | n/a | Profound atrophy of small fibers, large fibers and neurogenic muscle | 6 x myopathic features, diffuse variation in fiber size, increased internal and central nuclei, clustering of regenerating fibers | n/a | n/a | Rare atrophic fibers, increased internal nuclei |
| **Brain MRI**  **Abnormalities** | Polymicrogyria, dysmorphic basal ganglia, cerebellar dysplasia, white matter changes | T2 hyperintensities | n/a | n/a | White matter changes (1) | n/a | n/a | n/a | n/a | Signal change in globus pallidus (1); small cerebellum (1) (2/6 total) | Linear calcification in frontal lobe (1/4) | n/a | Normal Brain MRI |
| **Liver transaminases** | Normal | n/a | n/a | Elevated | Elevated (11/11) | n/a | Elevated | + (3/6) | n/a | n/a | n/a | n/a | n/a |
| **Serum lactate** | - (0.6, 1.1) | n/a | n/a | Normal | + (9/9) | n/a | n/a | n/a | n/a | n/a | n/a | Normal | Normal |
| **Elevated CK**  **levels (IU/L)** | + (2453- 2755) | + (600-3,100) | n/a | + | Increased (11/11) | + | Increased | + (3/6) | + (5532) | + (11/11)  (300-12,000 CK) | + (4/4)  (800-9000 CK) | 497 / 2,067 | >2000 |
| **Others** | Encephalopathy | Walked at 2.5yo | Intestinal malrotation |  | VSD x 2  Calf Muscle Hypertrophy  Splenomegaly  Low Immune Cell Counts | Non-specified skeletal abnormalities |  | Individuals with VSD, hypoglycemia, calf muscle hypertrophy | Clinodactyly, negative proprioceptive reflex |  |  |  |  |

**Supplemental Table 1 : Reported Cases, Genotypes and Phenotypes of MPXPS Secondary to Pathogenic Variants in MICU1.** OA – Optic atrophy, CTS – cataracts, NYS – nystagmus, PTS – ptosis, HMP – hypermetropia, ASM – Astigmatism, n/a – not available, or not reported
